# A study on the prediction of targeted therapy efficacy in advanced lung adenocarcinoma patients with EGFR mutations using CT-based delta-radiomics model

**DOI:** 10.3389/fmed.2025.1599206

**Published:** 2025-05-30

**Authors:** Zekai Wu, Peiyan Hua, Xiuying Chen, Jie Lei, Laian Zhang, Peng Zhang

**Affiliations:** ^1^Department of Radiology, The Second Hospital of Jilin University, Changchun, China; ^2^Department of Radiology, Jiangyou People’s Hospital, Mianyang, China; ^3^Department of Thoracic Surgery, The Second Hospital of Jilin University, Changchun, China; ^4^Department of Radiology, The FAW General Hospital of Jilin Province, Changchun, China

**Keywords:** lung adenocarcinoma, EGFR, targeted therapy, radiomics, delta-radiomics, nomogram

## Abstract

**Objective:**

This study aimed to evaluate the predictive performance of integrated clinical and CT-based radiomic models for assessing targeted therapy efficacy in advanced lung adenocarcinoma patients with EGFR (epidermal growth factor receptor) mutations.

**Materials and methods:**

This retrospective study included 106 EGFR-mutated advanced lung adenocarcinoma patients treated with targeted therapies at the Second Hospital of Jilin University (2020–2023). Patients were classified as responders (PR) or non-responders (SD/PD) based on RECIST (Response Evaluation Criteria in Solid Tumors) 1.1 criteria, then randomly divided into training (*n* = 74) and validation (*n* = 32) cohorts at a 7:3 ratio. We segmented tumor regions on pre-and post-treatment CT scans using ITK-SNAP, then extracted radiomic features and applied mRMR-LASSO (Minimum Redundancy Maximum Relevance–Least Absolute Shrinkage and Selection Operator). A delta-radiomics model was developed by quantifying feature changes between treatment phases. Significant clinical predictors identified by logistic regression were integrated with radiomic features to build a combined model. Performance was assessed via AUC, sensitivity, specificity, accuracy, positive predictive value (PPV), negative predictive value (NPV), DeLong’s test, calibration curves, and decision curve analysis.

**Results:**

In the pre-treatment radiomics model, the AUC, accuracy, sensitivity, specificity, PPV, and NPV of the training cohorts were 0.751, 0.690, 0.737, 0.639, 0.683, and 0.697; in validation cohorts, these values were 0.726, 0.656, 0.778, 0.500, 0.667, and 0.636. In the delta-radiomics model, the AUC, accuracy, sensitivity, specificity, PPV, and NPV of the training cohorts were 0.906, 0.865, 0.868, 0.861, 0.868, and 0.861, vs. 0.825, 0.719, 0.722, 0.714, 0.765, and 0.667 in validation. For the clinical model, the AUC, accuracy, sensitivity, specificity, PPV, and NPV of the training cohorts were 0.828, 0.729, 0.737, 0.722, 0.737, and 0.722, compared to 0.766, 0.750, 0.722, 0.786, 0.812, and 0.688 in validation. In the combined model, the AUC, accuracy, sensitivity, specificity, PPV, and NPV of the training cohorts were 0.977, 0.946, 0.947, 0.944, 0.947, and 0.944, while in the validation cohorts, these values were 0.913, 0.781, 0.778, 0.786, 0.824, and 0.733.

**Conclusion:**

The combined model integrating delta-radiomics with clinical predictors demonstrates superior predictive performance for evaluating targeted therapy efficacy in EGFR-mutated advanced lung adenocarcinoma, significantly outperforming conventional radiomics models relying exclusively on pre-treatment imaging data.

## 1 Introduction

Lung cancer is one of the most prevalent cancers worldwide, characterized by high incidence and mortality rates. Global estimates project projected to result in approximately 1.25 million deaths in 2024, accounting for nearly 20% of all cancer mortality and maintaining its position as the leading cause of cancer death in both genders (1–3). The absence of specific early symptoms often leads to delayed diagnosis, with many lung cancer patients already presenting lymph node involvement or distant metastases at initial detection, rendering curative surgical intervention unfeasible (4–14). Smoking is the primary risk factor for lung cancer, but exposure to second-hand smoke, family history, and carcinogenic chemicals also contribute to the risk of developing the disease (5). Lung cancer is categorized into two primary types: non-small cell lung cancer (NSCLC) and small cell lung cancer, with NSCLC being more prevalent. Non-small cell lung cancer (NSCLC) comprises three subtypes: adenocarcinoma, squamous cell carcinoma, and giant cell carcinoma, with adenocarcinoma being the predominant pathological type, representing 40% of all lung cancer cases (7, 8). Surgical intervention is frequently employed to address early-stage lung cancer, but individuals with advanced-stage lung cancer typically exhibit poor responses to surgical treatment, this enabled the researchers to investigate alternative treatment modalities, including radiation, chemotherapy, and immunotherapy. In recent years, molecular targeted therapy has significantly improved in non-small cell lung cancer, offering enhanced alternatives for clinical diagnosis and treatment, particularly for advanced lung adenocarcinoma and other lung cancer variants with adenocarcinoma components, EGFR tyrosine kinase inhibitors (EGFR-TKIs) have emerged as the primary pharmacological agents for the management of intermediate and advanced non-small cell lung cancer, these novel targeted medicines not only markedly enhanced clinical results but also prolonged patient survival (9, 11, 12).

The RECIST is the main way to judge how well targeted therapies work in solid tumors (13, 14). However, it primarily focuses on tumor size changes and fails to capture tumor biological behavior or microenvironmental alterations. Radiomics technology provides a novel approach for disease analysis and clinical prediction through extracting high-dimensional quantitative features from imaging modalities such as CT and MRI (15, 16). Unlike RECIST, radiomics can reveal intricate texture features, both within tumors and at their peripheries, that are not readily observable, this is crucial for understanding disease dynamics and evaluating the effectiveness of treatment (17). Delta-radiomics (a subset of radiomics) analyzes temporal feature changes from longitudinal imaging using deep learning algorithms to enhance treatment outcome prediction accuracy (18, 19). Fan et al. (20) developed a nomogram integrating delta-radiomics features with clinical parameters, which accurately predicted a complete pathological response in patients with esophageal squamous cell carcinoma following neoadjuvant chemoradiotherapy; this model showed high accuracy and stability in both training and validation cohorts. By analyzing imaging changes in advanced lung adenocarcinoma patients before and after targeted therapy, this study aims to develop radiomics models to establish scientific evidence for optimizing treatment strategies in this population. This study innovatively integrates delta-radiomics (dynamic tumor changes) with clinical predictors, offering a multi-dimensional predictive framework.

## 2 Materials and methods

### 2.1 Patient characteristics

In this retrospective study, we collected data on 106 patients with pathologically confirmed advanced lung adenocarcinoma admitted to the Second Hospital of Jilin University between January 2020 and June 2023, all of whom were confirmed to have EGFR mutations. Pathological specimens were obtained via CT-guided biopsy or bronchoscopy. EGFR mutations were confirmed through next-generation sequencing (NGS). We obtained CT images from these patients at initial admission, prior to targeted therapy, and after 2 to 3 cycles of treatment. Treatment response was categorized according to RECIST 1.1 guidelines, with responders defined as achieving partial response (PR, *n* = 56) and non-responders comprising stable disease (SD) or progressive disease (PD, *n* = 50). Subsequently, patients were randomly divided into training (*n* = 74) and validation (*n* = 32) cohorts at a 7:3 ratio using a stratified randomization method to ensure balanced distributions of PR and SD/PD groups between cohorts. The study protocol was approved by the hospital’s Institutional Review Board with waiver of informed consent. The inclusion criteria were as follows: (1) Histopathologically confirmed lung adenocarcinoma; (2) EGFR mutation confirmed by molecular testing; (3) Stage III-IV disease per 8th edition TNM staging treated with targeted therapy alone; (4) Availability of complete pre−/post-treatment CT imaging data; (5) Complete medical records; (6) No history of other malignancies. The exclusion criteria were as follows: (1) Absence of follow-up CT imaging after 2–3 treatment cycles; (2) Poor image quality or inability to accurately assess lesion boundaries; (3) Receipt of non-targeted antitumor therapies (e.g., radiotherapy or chemotherapy) during treatment; (4) Incomplete clinical data or missing key parameters.

### 2.2 CT images acquisition

All participants underwent standardized pre-and post-treatment thoracic CT examinations using 256-slice multidetector CT systems (Philips Brilliance CT and GE Revolution CT). Prior to imaging acquisition, standardized respiratory training was conducted to ensure consistent breath-holding at full inspiration in the supine position, thereby minimizing respiratory motion artifacts. The scan range extended from the thoracic apex to the diaphragmatic level, covering the entire lung parenchyma. The scanning parameters for the Philips Brilliance CT were set as follows: slice thickness and interval of 5 mm, tube voltage of 120 kVp, tube current of 250 mAs, pitch ratio of 0.575, and matrix size of 512 × 512. The Revolution CT settings comprised a slice thickness and interval of 5 mm, a tube voltage of 140 kVp, a tube current of Smart mAs, pitch ratio of 1, and matrix size of 512 × 512. All raw datasets were reconstructed into 1-mm-thin slices using multiplanar reconstruction (MPR) algorithms on dedicated imaging workstations, with final volumetric data stored in NII format for quantitative radiomic analysis. This optimized imaging protocol ensures high spatial resolution and reproducibility, providing reliable technical support for longitudinal oncological evaluations.

### 2.3 Patient clinical and imaging data

We retrieved clinical and imaging data from the study’s patients from the hospital’s electronic medical record system. The dataset included patient demographics such as gender and age, smoking history, TNM staging, and maximum tumor diameters prior to and following treatment. The TNM staging employed the 8th edition of the TNM classification system for lung cancer, as established by the International Association for the Study of Lung Cancer (IASLC).

### 2.4 ROI segmentation

**Figure 1:**
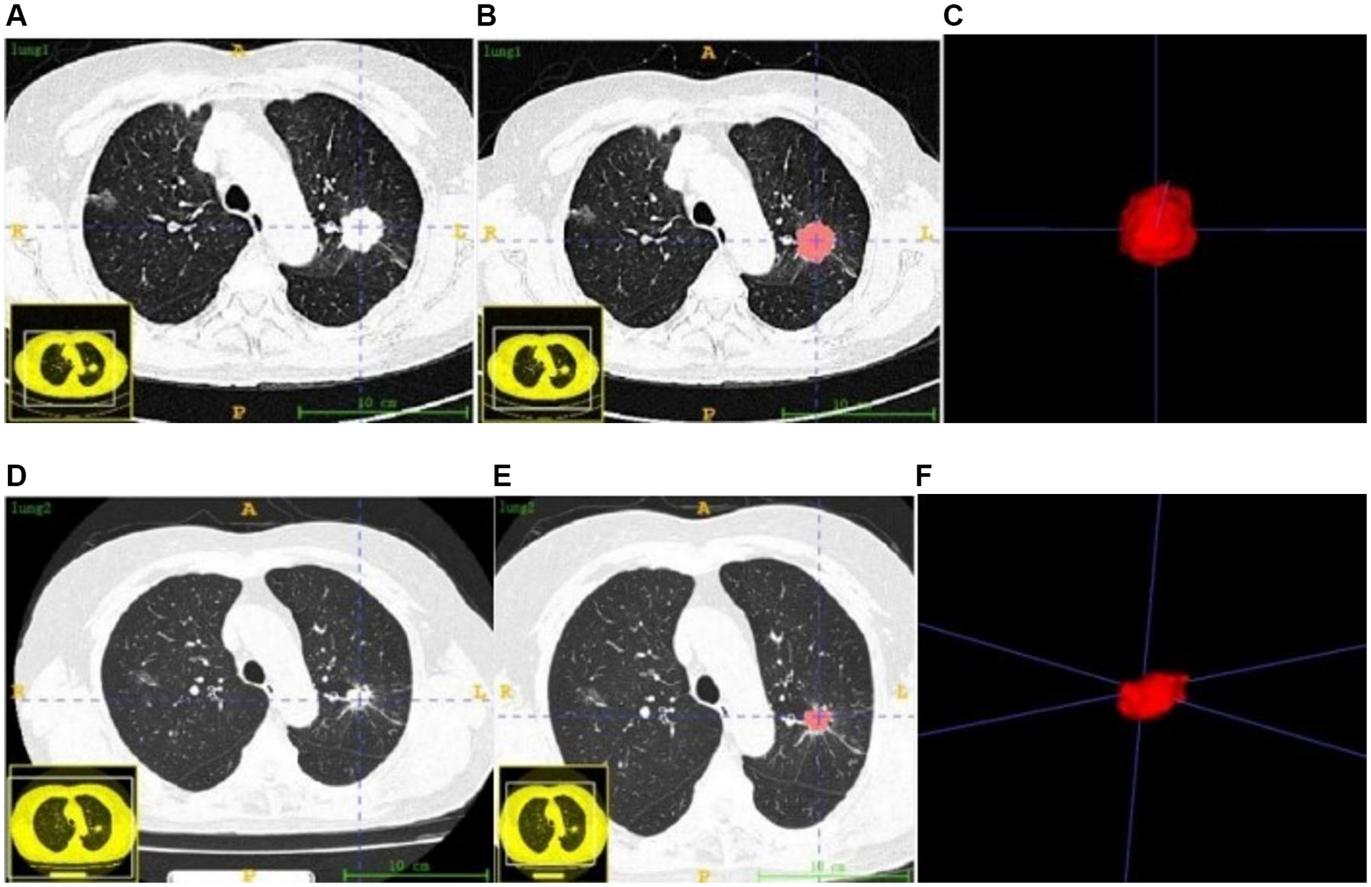
Tumor ROI before targeted therapy **(A–C)** and after targeted therapy **(D–F)**.

This study employed images acquired from two distinct CT scanners. To deal with potential variations in scanning parameters, we preprocessed the images by means of grayscale discretization, normalization, and resampling before using FeAture Explorer Pro (FAE, version 0.5.8) for feature extraction. Experienced physicians manually delineated tumor regions of interest using ITK-SNAP (v.3.8.0)^1^ software (Figure 1). A radiologist with 5 years of experience carried out semi-automatic delineation, ensuing comprehensive inclusion of the lesion while maintaining adjacent structures. To assess the inter-observer reproducibility of feature extraction, a radiologist with a decade of experience re-evaluated the images of 50 randomly selected patients. The reliability of the features was quantified using the intraclass correlation coefficient (ICC), with features demonstrating ICC ≥ 0.75 retained for model construction. Discrepancies identified during the evaluation process were resolved through consultation with a senior physician. All data were subsequently stored in NII format. Throughout this process, physicians remained unaware of patient clinical data and treatment outcomes to ensure objectivity in their assessments.

### 2.5 Feature extraction and selection

Prior to feature extraction, we standardized all CT images. We then employed FeAture Explorer Pro (FAE, version 0.5.8) to conduct high-throughput radiomics feature extraction on the CT images of patients with lung cancer. We organized the extracted raw image features into four primary categories: first-order features, shape-based features, gray-level co-occurrence matrix (GLCM), and gray-level run length matrix (GLRLM). Subsequently, we quantified the radiomics features before and after targeted therapy to calculate their net changes (defined as the post-treatment feature values minus baseline values). The delta-radiomics features (ΔRFs) were computed using the formula: ΔRFs = Feature (TP0) - Feature (TP1), where TP0 and TP1 represent pre-and post-treatment time points, respectively.

All extracted features were z-score normalized. Subsequently, intraclass correlation coefficient (ICC) analysis was performed to assess inter-observer consistency, with features demonstrating ICC values ≥0.75 retained for subsequent analysis. The minimum Redundancy Maximum Relevance (mRMR) algorithm was then employed to reduce feature redundancy. Finally, a Least Absolute Shrinkage and Selection Operator (LASSO) regression model was applied to identify optimal predictors. This rigorous multi-stage feature selection pipeline establishes a methodological foundation for constructing robust radiomics prediction models.

### 2.6 Model construction and validation

In this study, we employed the minimum redundancy maximum relevance (mRMR) algorithm in conjunction with the least absolute shrinkage and selection operator (LASSO) to identify salient features. The mRMR algorithm was utilized to select features that exhibit strong associations with tumor treatment response while minimizing redundancy and inter-feature correlation, thereby reducing the risk of overfitting. The LASSO technique further refined the feature set by identifying features with non-zero coefficients. The optimal regularization parameter (*λ*) for LASSO was selected via 10-fold cross-validation by identifying the value that minimized the binomial deviance from a predefined range of λ, features with non-zero coefficients under this optimal λ were retained for model construction. The selected features were then used to compute the radiomics score (Radscore), which is derived from the sum of the products of each feature and its corresponding regression coefficient, along with an intercept term. The formula for Radscore is: Radscore = *Σ*(αi * Xi) + *β*, where αi denotes the regression coefficient, Xi represents the feature value, and β is the intercept. This methodology yielded a quantitative radiomics score for each patient, indicating the expected response to targeted therapy.

To validate the predictive performance of radiomic models before and after targeted therapy, we applied logistic regression with 10-fold cross-validation repeated 10 times. Initially, univariate logistic regression was applied to clinical and imaging data to screen for significant variables, which were subsequently incorporated into multivariate logistic regression to identify key predictive features associated with the response to EGFR mutation-targeted therapy in patients with advanced lung adenocarcinoma. Based on these variables, we constructed a clinical prediction model. Finally, we integrated the clinical predictive features with radiomics features to develop a combined model and established a radiomics nomogram for enhanced intuitive prediction.

### 2.7 Statistical analysis

All statistical analyses were performed using IBM SPSS 25.0 and R 4.2.2. We performed a normality test using the Kolmogorov–Smirnov test, representing standard measurements of a normal distribution as Mean ± SD, and using the independent sample t test for inter-group comparisons. Categorical variables are expressed as frequency (rate) and compared between groups using Chi-square tests or Fisher precision tests. A two-tailed test with a *p* < 0.05 (typically ≤ 0.05) was indicative of a statistically significant difference.

## 3 Results

### 3.1 Clinical model

Univariate logistic regression analysis of pertinent clinical characteristics and CT imaging findings revealed significant predictors of targeted therapeutic success, including patient gender, smoking history, and the greatest tumor diameter post-treatment (*p* < 0.05). Table 1 details these significant predictors. Multivariate logistic regression analysis identified smoking history and post-treatment maximum tumor diameter as independent predictors. Table 2 details the multivariate results, which were integrated into the clinical model. Table 3 shows the clinical model performance: In the training cohort, the model achieved an AUC of 0.828 (95% CI: 0.734–0.922) with accuracy 0.729, sensitivity 0.737, specificity 0.722, PPV 0.737, and NPV 0.722. The validation cohort showed an AUC of 0.766 (95% CI: 0.584–0.948) with accuracy 0.750, sensitivity 0.722, specificity 0.786, PPV 0.812, and NPV 0.688.

**Table 1:** Univariate logistic regression analysis of clinical and CT imaging results.

Characteristics	PR(n=50)	PD/SD(n=56)	*p*	
			Training cohort	Validation cohort
Age,(mean±SD)	62.66±7.52	59.14±8.49	0.100	0.153
Sex,n (%)			0.037*	0.419
Male	23(46.00%)	33(58.93%)		
Female	27(54.00%)	23(41.07%)		
T			0.444	0.590
1	14(28.00%)	12(21.43%)		
2	19(38.00%)	25(44.64%)		
3	10(20.00%)	11(19.64%)		
4	7(14.00%)	8(14.29%)		
N			0.248	0.843
0	25(50.00%)	20(35.71%)		
1	4(8.00%)	6(10.71%)		
2	11(22.00%)	21(37.5%)		
3	10(20.00%)	9(16.07%)		
M			0.099	0.341
0	22(44.00%)	15(26.79%)		
1	28(56.00%)	41(73.21%)		
Pre-treatment Max Diameter Median(Q1, Q3)	38.00(25.00,49.50)	39.50(29.50,52.50)	0.446	0.287
Post-treatment Max Diameter Median(Q1, Q3)	27.00(20.00,40.00)	30.50(17.50,39.00)	<0.001*	0.114
Smoking			0.042*	0.290
No	42(84.00%)	36(64.29%)		
Yes	8(16.00%)	20(35.71%)		

Mean ± SD means mean± standard deviation, n means quantity; Median (Q1,Q3) represents the median (upper and lower quartiles). *indicates statistical significance (*p* < 0.05).

**Table 2:** Multivariate logisitic regression analysis of clinical and CT imaging data.

Variable	B	S.E.	Wald	df	*p*	OR	95% CI	
							Lower Limit	Upper Limit
Sex	−0.991	0.582	2.901	1	0.089	0.371	0.119	1.161
Max diameter after treatment	−0.094	0.025	13.885	1	<0.001*	0.910	0.866	0.956
Smoking	2.756	0.946	8.480	1	0.004*	15.738	2.462	26.591

*indicates statistical significance (*p* < 0.05).

**Table 3:** Performance comparison of different models in training and validation.

Model	Cohort	Sensitivity	Specificity	AUC	95%CI	PPV	NPV	Accuracy
Pre-Radscore	Training cohort	0.737	0.639	0.751	(0.639,0.864)	0.683	0.697	0.690
	Validation cohort	0.778	0.500	0.726	(0.539,0.913)	0.667	0.636	0.656
Delta-Radscore	Training cohort	0.868	0.861	0.906	(0.826,0.987)	0.868	0.861	0.865
	Validation cohort	0.722	0.714	0.825	(0.681,0.970)	0.765	0.667	0.719
Clinic model	Training cohort	0.737	0.722	0.828	(0.734,0.922)	0.737	0.722	0.729
	Validation cohort	0.722	0.786	0.766	(0.584,0.948)	0.812	0.688	0.750
Combined model	Training cohort	0.947	0.944	0.977	(0.938,1.000)	0.947	0.944	0.946
	Validation cohort	0.778	0.786	0.913	(0.818,1.000)	0.824	0.733	0.781

### 3.2 Radiomics models

**Figure 2:**
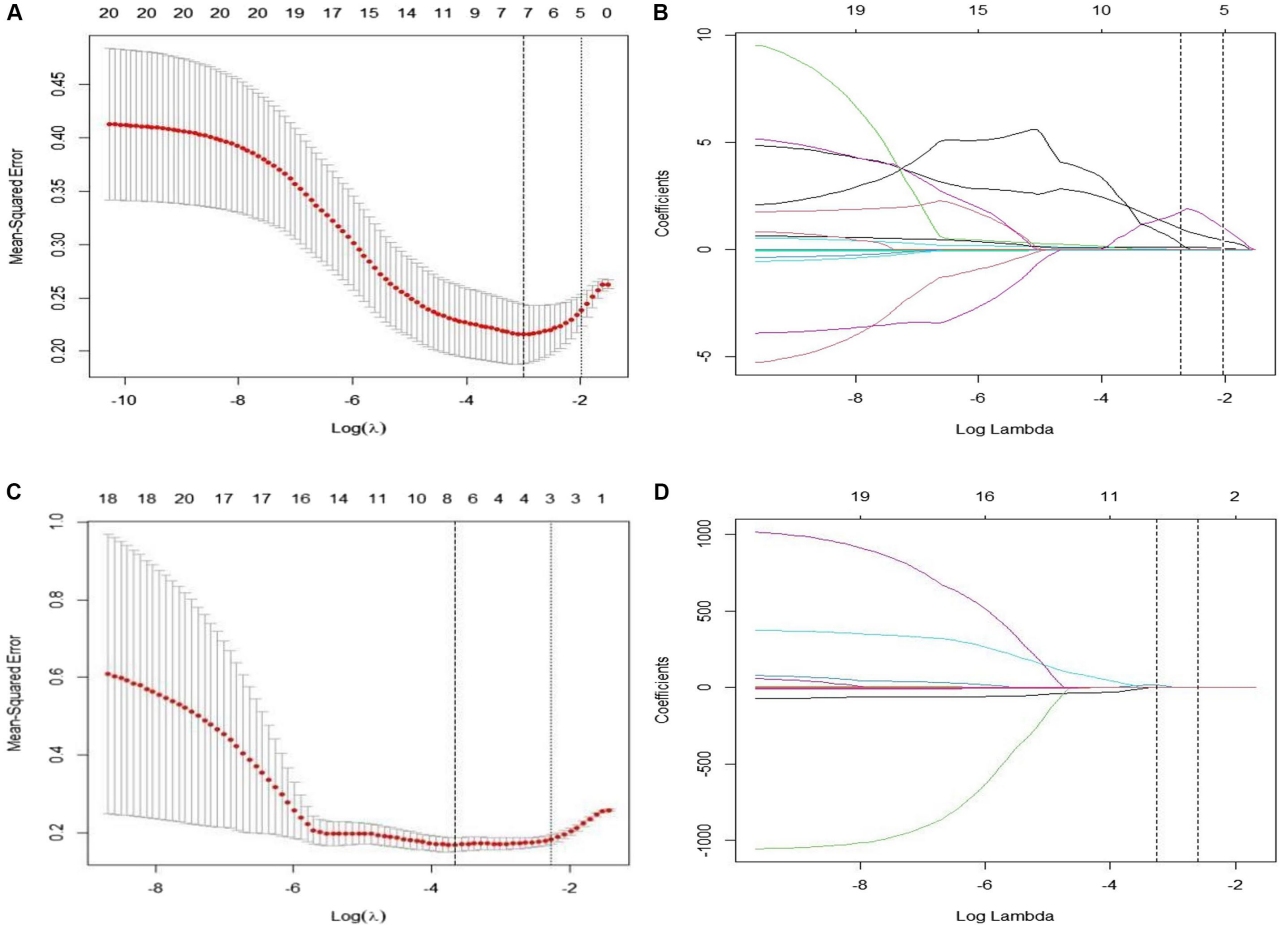
**(A–D)** Show the results of the 10-fold cross-validation method and LASSO regression analysis, respectively, removing highly redundant features to obtain the optimal features.

In this study, we initially employed the Maximum Relevance Minimum Redundancy (mRMR) algorithm to perform preliminary feature selection on CT imaging data from 106 patients with EGFR-mutant advanced lung adenocarcinoma. This step aimed to identify radiomic features most closely associated with the efficacy of targeted therapy. Subsequently, we applied the Least Absolute Shrinkage and Selection Operator (LASSO) algorithm to these preliminarily selected features and utilized a 10-fold cross-validation method to filter out statistically significant features (non-zero coefficients). This process ultimately identified eight key radiomic features that were used to construct a radiomic signature for predicting treatment outcomes. Among these eight features, five were derived from pre-treatment CT scans, while three were based on difference characteristics between pre-and post-treatment images. Detailed results of feature selection along with corresponding imaging interpretations are illustrated in Figure 2.

The characteristics retrieved before treatment were designated as original_shape_LeastAxisLength, original_shape_MinorAxisLength, original_shape_SurfaceVolumeRatio, wavelet-HLL_firstorder_Uniformity and wavelet-HHL_glcm_DifferenceVariance. The features were utilized to compute the radiomics label scores, and a logistic regression approach was applied to develop the pre-treatment radiomics model (RS-Pre-treatment). The Radscore formula for this model is outlined as follows: Pre-Radscore = −0.2735651210–006857017 × original_shape_LeastAxisLength-0.020428557 × original_shape_MinorAxisLength+0.395147962 × original_shape_SurfaceVolumeRatio+0.054076360 × waveletHLL_firstorder_Uniformity+0.927680463 × wavelet-HHL_glcm_DifferenceVariance.

The delta-radiomics features retrieved from pre and post-treatment changes included original_shape_sphericity, original_firstorder_median, and wavelet-LHH_glrlm_HighGrayLevelRunEmphasis. The characteristics were employed to compute the radiomics label scores, and logistic regression was utilized to develop the radiomics model (RS-Delta). The Radscore equation for this model is as follows:

Delta-Radscore = −5.049048504 + 4.903682586 × original_shape_Sphericity–0.001830174 × original_firstorder_Median + 0.009943380 × wavelet-LHH_glrlm_HighGrayLevelRunEmphasis.

Table 3 shows the performance of the pre-treatment Radscore model: In the training cohort, the model achieved an AUC of 0.751 (95% CI: 0.639–0.864) with accuracy of 0.690, specificity of 0.639 and sensitivity of 0.737, positive predictive value (PPV) of 0.683 and negative predictive value (NPV) of 0.697. In the validation cohort, the model showed an AUC of 0.726 (95% CI: 0.539–0.913) with accuracy of 0.656, specificity of 0.500 and sensitivity of 0.778, PPV of 0.667 and NPV of 0.636. For the Delta-Radscore model, in the training cohort, it achieved an AUC of 0.906 (95% CI: 0.826–0.987) with accuracy of 0.865, specificity of 0.861 and sensitivity of 0.868, PPV of 0.868 and NPV of 0.861. In the validation cohort, the model demonstrated an AUC of 0.825 (95% CI: 0.681–0.970) with accuracy of 0.719, specificity of 0.714 and sensitivity of 0.722, PPV of 0.765 and NPV of 0.667.

### 3.3 Combined model

The combined model incorporated three key predictors: post-treatment maximum tumor diameter, smoking history, and radiomics signatures. The combined model demonstrated robust diagnostic performance, achieving an AUC of 0.977 (95% CI: 0.938–1.000) in the training cohort with accuracy 0.946, sensitivity 0.947, specificity 0.944, PPV 0.947, and NPV 0.944. In the validation cohort, the model maintained strong performance at an AUC of 0.913 (95% CI: 0.818–1.000) along with accuracy 0.781, sensitivity 0.778, specificity 0.786, PPV 0.824, and NPV 0.733. Table 3 comprehensively details these performance metrics.

**Figure 3:**
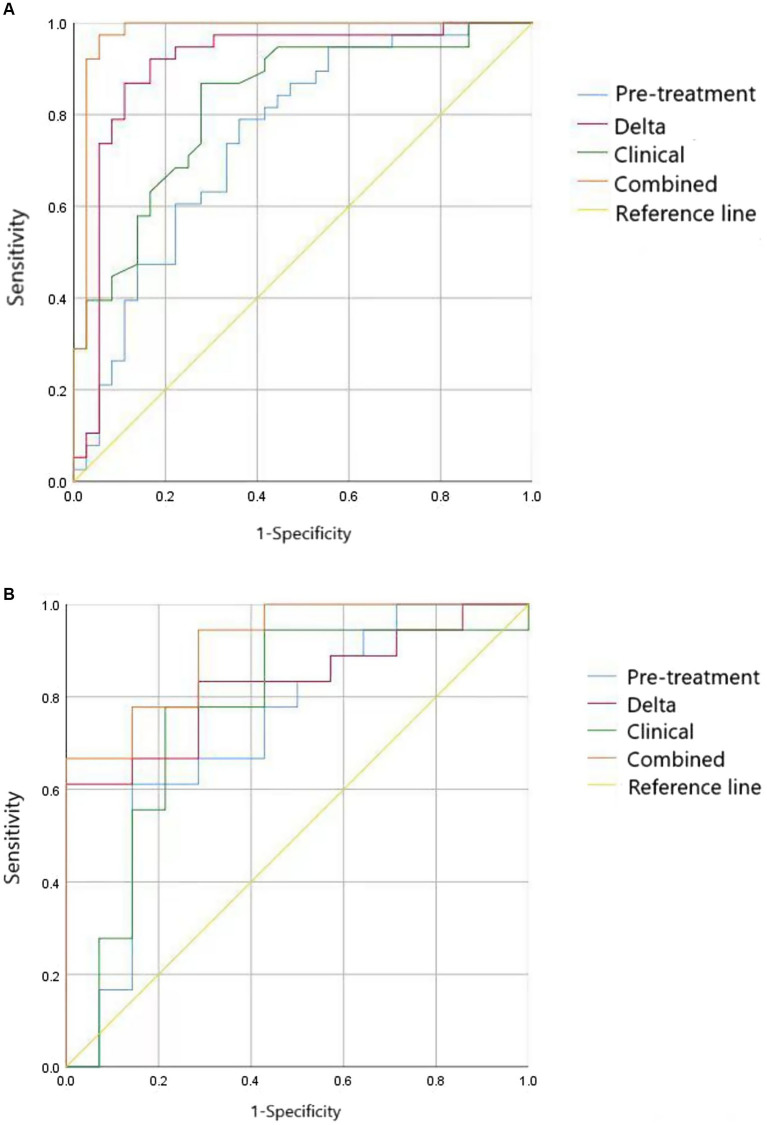
ROC curve of four models (clinical, pre-treatment, delta, combined) in the training **(A)** and validation cohorts **(B)**.

Comparative analysis using DeLong’s test revealed statistically significant differences in receiver operating characteristic (ROC) curves between models (Figure 3). Table 4 summarizes the statistical outcomes of all model comparisons. In the training cohort, the integrated model exhibited superior predictive performance compared to both the clinical model (*p* < 0.05) and radiomics models based on pre-treatment features (pre-Radscore) or delta-radiomics features (delta-Radscore). Within the validation cohort, significant differences were observed between the integrated model and pre-Radscore model (*p* < 0.05), as well as between pre-Radscore and Delta-Radscore models (*p* < 0.05). Other model comparisons did not achieve statistical significance. In addition, we observed differences in performance between the training and validation cohorts, particularly in models such as delta-radiomics, where higher accuracy in the training cohort (AUC: 0.906) decreased in validation (AUC: 0.825). This pattern suggests potential overfitting, where models may over-adapt to training data nuances rather than generalizable patterns.

**Table 4:** DeLong’s test.

Cohort		Combined model	Clinical model	Pre-Radscore
Training cohort	Clinical model	0.003*		
	Pre-Radscore	<0.001*	0.302	
	Delta-Radscore	0.039*	0.232	0.027*
Validation cohort	Clinical model	0.113		
	Pre-Radscore	0.041*	0.768	
	Delta-Radscore	0.151	0.654	0.404

*Indicates that the difference is significant.

### 3.4 Construction and evaluation of nomograms

**Figure 4:**
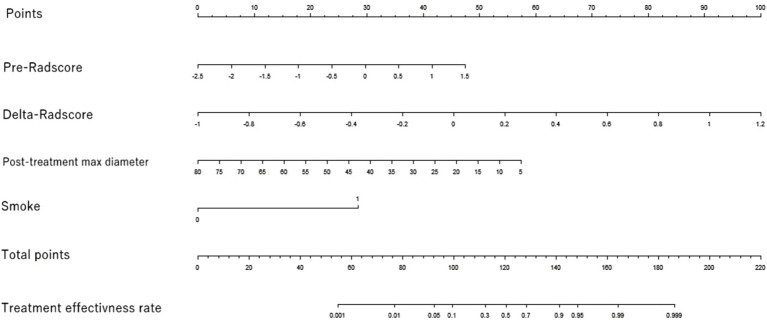
Nomogram of the efficacy prediction model. The nomogram combines two separate imaging predictors (max diameter after treatment and smoking history) with radiomics features based on the pre-radscore and the delta-radscore.

**Figure 5:**
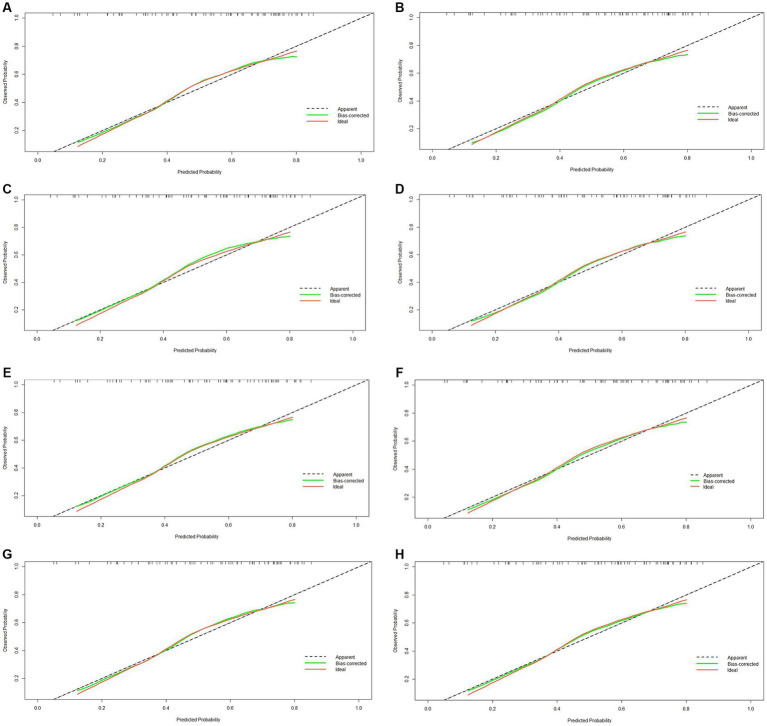
Calibration curve of four models (clinical, pre-treatment, delta, combined) in the training **(A–D)** and validation cohorts **(E–H)**.

**Figure 6:**
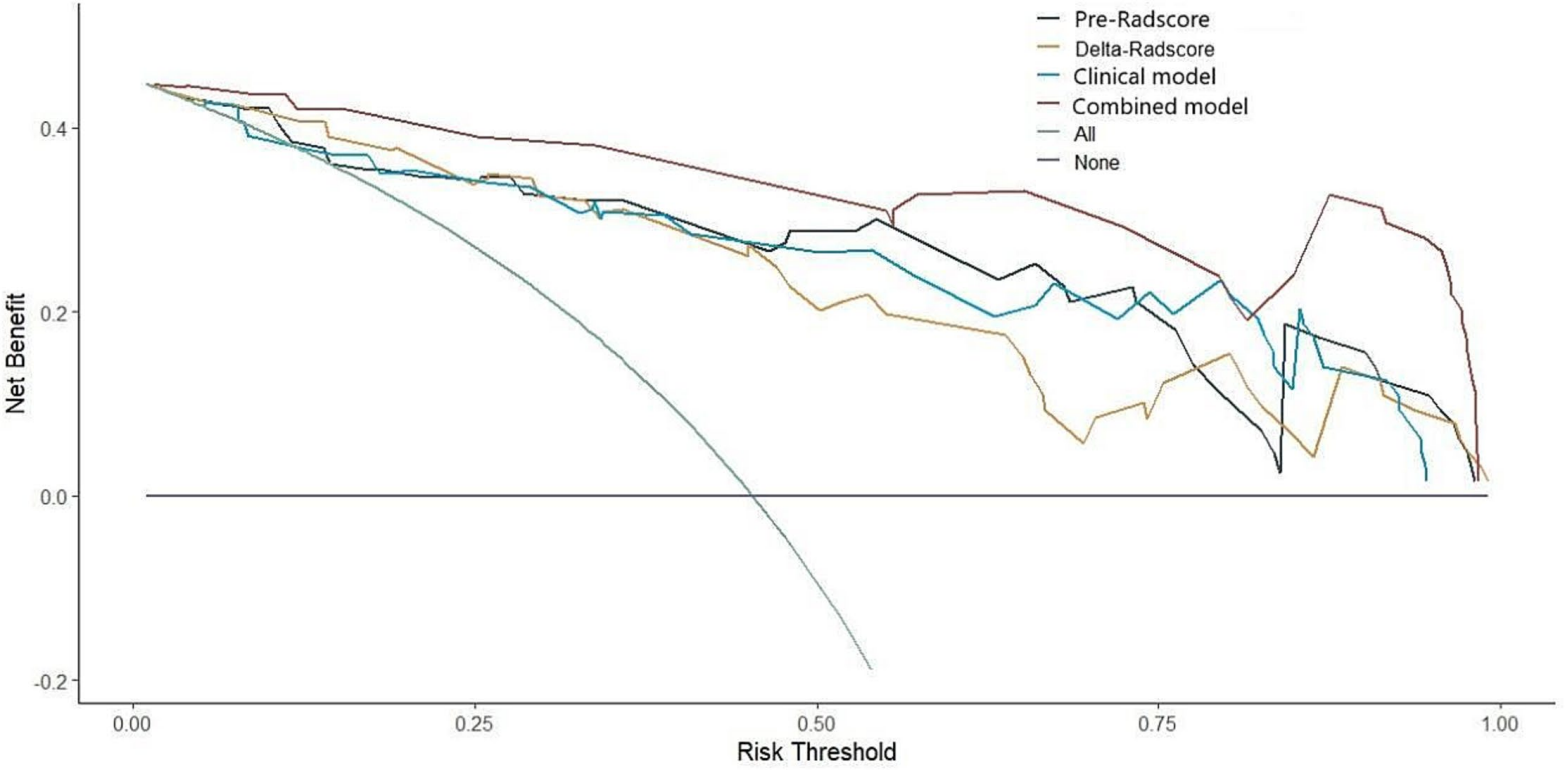
Decision curve of four models (clinical, pre-treatment, delta, combined).

This study developed a nomogram based on the combined model (Figure 4). Calibration curves were generated for the clinical model, radiomics model (incorporating pre-treatment and delta features), and combined model (Figure 5). These calibration plots demonstrated satisfactory performance in both cohorts, with all models closely aligning with the ideal 45° reference line, indicating strong agreement between predicted probabilities and observed outcomes. The Hosmer-Lemeshow test showed non-significant results (*p* > 0.05) for both pre-treatment and delta-feature-based radiomics models, confirming no statistically significant deviations from actual observations. Decision curve analysis demonstrated that the combined model provided higher net benefits than other models across clinically relevant threshold probabilities, offering evidence-based guidance for clinical decisions (Figure 6).

## 4 Discussion and conclusion

Molecular targeted therapy effectively inhibits cancer progression by targeting specific molecular pathways, demonstrating clinical success in malignancies including breast cancer, leukemia, colorectal cancer, lung cancer, and ovarian cancer (21). Its improved safety profile compared to conventional chemotherapy has driven widespread adoption, particularly for advanced lung adenocarcinoma (22). In clinical practice, therapeutic efficacy assessment primarily relies on RECIST criteria via imaging (13, 23). However, tumor volume changes often lag behind microenvironmental heterogeneity. Radiomics addresses this limitation by quantifying early microstructural alterations through medical image analysis. Delta radiomics, a specialized subset, tracks longitudinal tumor characteristic changes, providing dynamic response monitoring. Nardone et al. (24) conducted a systematic review of the research trajectories of Delta radiomics across various domains, including preclinical and methodological studies, as well as applications in multiple cancer types, such as lung, colorectal, prostate, and gastrointestinal cancers. Barabino et al. (25) showed that delta-radiomics outperforms RECIST in early immunotherapy response assessment for NSCLC. Their analysis identified key CT-derived features (including markers of pseudo-progression) as quantitative biomarkers to address RECIST limitations in heterogeneous tumor behavior. Our results demonstrate that delta radiomics provides significant clinical value in oncology, including applications in differential diagnosis, prognosis, treatment response prediction, and adverse event assessment.

This research examines the predictive effectiveness of targeted therapy in patients with advanced lung adenocarcinoma possessing EGFR mutations. Aerts et al. (26) analyzed pre-and post-gefitinib CT scans in 47 NSCLC patients, identifying 13 radiomic features, among which the pretreatment Laws-Energy feature demonstrated superior prediction of EGFR mutation status compared to traditional size measurements (AUC 0.74–0.91), while feature changes across scans reliably indicated treatment response. Additionally, our study is the first to investigate imaging feature alterations in NSCLC patients with different EGFR mutation statuses before and after targeted therapy. We applied the mRMR algorithm to identify strongly correlated and non-redundant features, followed by LASSO optimization with L1 regularization, ultimately extracting eight effective features. Prior to dimensionality reduction, wavelet transform captured multi-scale directional texture information, generating comprehensive feature sets for analysis. Tang (27) demonstrated that the wavelet transform, which integrates features from both the wavelet domain and the original CT domain, offers considerable benefits in classifying hepatocellular carcinoma (HCC) and other liver lesions, thereby improving tumor classification performance significantly. Wu et al. (28) demonstrated the predictive value of wavelet transform features for tumor histology, with most features showing discriminative power. The feature “Wavelet_HLH_glcm_correl1” outperformed others in univariate analysis, achieving the highest AUC of 0.66. Our findings confirm wavelet transform’s utility in extracting radiomic features for tumor classification, particularly in CT-based lung cancer subtyping. As a widely used texture analysis method, wavelet-derived features exhibit distinct advantages over conventional approaches. The wavelet transform enhances image detail and elucidates the internal characteristics of tumors, as well as accurately delineating their edges, this information contributes to the understanding of tumor development patterns and their potential prognostic implications (29, 30).

The findings of this study indicate that the integration of clinical, pretreatment, and Delta features achieved an AUC of 0.977 (95% CI: 0.938–1.000) in the training group and 0.913 (95% CI: 0.818–1.000) in the validation group, surpassing the performance of all other models evaluated. Furthermore, the accuracy, sensitivity, and specificity in both groups were superior to those observed with alternative models. Wang et al. (31) investigated early predictions of immune treatment responses in patients with metastatic melanoma undergoing anti-PD-1 and CTLA-4 therapies by developing several radiomics models through analysis of treatment-related and Delta features. The combined model demonstrated superior predictive accuracy for immunotherapy response compared to single-feature models, attributable to its incorporation of clinical parameters, pretreatment imaging features, and treatment-induced tumor dynamics. By synergistically analyzing interactions among these factors (e.g., clinical-imaging correlations), the model provides a comprehensive framework for early efficacy assessment, enhancing both interpretability and precision.

Lin et al. (32) conducted a retrospective analysis of 191 high-grade osteosarcoma patients receiving neoadjuvant chemotherapy, developing a CT-based delta-radiomics model using 540 radiomic features from pre-and post-treatment CT images. This model demonstrated superior AUC values compared to single-timepoint radiomics in both training and validation cohorts. Similarly, Li et al. (33) established delta-radiomics models to identify early EGFR-TKI resistance in lung adenocarcinoma, incorporating baseline, first follow-up, and final pre-progression imaging. Their delta-models consistently outperformed conventional radiomics across training and validation groups. In this study, the delta-radiomics model demonstrated higher accuracy, sensitivity, specificity, and AUC than the pretreatment model in both training and validation cohorts. However, DeLong tests revealed statistically significant differences between models only in the training group (*p* < 0.05), with no significance in the validation group (*p* > 0.05). These results conflict with findings from Lin (32) and Li (33) and deviate from expectations. We posit two explanations: (1) overfitting to the training data may restrict generalizable feature variation capture, or (2) limited validation cohort size/heterogeneity obscures true model differences. To address these limitations, future studies should: expand dataset diversity/size, integrate regularization with optimized feature selection, and validate performance through independent calibration cohorts.

This study advances understanding of advanced lung adenocarcinoma response to targeted therapy, but several limitations should be noted. First, as a single-center retrospective investigation, its limited sample size and inconsistent follow-up durations may affect result generalizability. Potential biases such as selection bias (e.g., exclusion of patients with incomplete imaging) and unmeasured confounders (e.g., variations in treatment adherence or genetic heterogeneity) should also be acknowledged. The semi-automatic segmentation method’s potential subjectivity may affect generalizability. While focused on targeted therapy responders, our findings require validation in broader clinical contexts encompassing chemotherapy and immunotherapy. Although currently limited to lung adenocarcinoma, future studies should encompass diverse lung cancer subtypes and treatment modalities to fully evaluate radiomics’ clinical utility. This study provides foundational insights, yet expanded multi-center research is imperative for validating feature applicability across therapeutic approaches.

In conclusion, the combined model, which integrates delta-radiomics with clinical predictors (e.g., post-treatment tumor size and smoking history), significantly improves the prediction of targeted therapy efficacy in advanced EGFR-mutated lung adenocarcinoma. This approach outperforms traditional radiomics models that rely exclusively on pre-treatment imaging, highlighting the value of dynamic imaging changes and clinical factors for personalized treatment assessment.

## Data Availability

The raw data supporting the conclusions of this article will be made available by the authors, without undue reservation.

## Footnotes

^1^ www.itksnap.org
